# UtroUp is a novel six zinc finger artificial transcription factor that recognises 18 base pairs of the utrophin promoter and efficiently drives utrophin upregulation

**DOI:** 10.1186/1471-2199-14-3

**Published:** 2013-01-30

**Authors:** Annalisa Onori, Cinzia Pisani, Georgios Strimpakos, Lucia Monaco, Elisabetta Mattei, Claudio Passananti, Nicoletta Corbi

**Affiliations:** 1 Institute of Molecular Biology and Pathology CNR, c/o Department of Molecular Medicine, University “Sapienza”, Viale Regina Elena 291, 00161, Rome, Italy; 2 Institute of Cell Biology and Neurobiology CNR, IRCCS Fondazione Santa Lucia, Via del Fosso di Fiorano 64, 00143, Rome, Italy; 3 Department of Physiology and Pharmacology, University Sapienza, Piazzale Aldo Moro 5, 00185, Rome, Italy

**Keywords:** DMD, Dystrophin, Utrophin, Zinc finger, Artificial transcription factor, Activation domain, Che-1/AATF

## Abstract

**Background:**

Duchenne muscular dystrophy (DMD) is the most common X-linked muscle degenerative disease and it is due to the absence of the cytoskeletal protein dystrophin. Currently there is no effective treatment for DMD. Among the different strategies for achieving a functional recovery of the dystrophic muscle, the upregulation of the dystrophin-related gene utrophin is becoming more and more feasible.

**Results:**

We have previously shown that the zinc finger-based artificial transcriptional factor “Jazz” corrects the dystrophic pathology in mdx mice by upregulating utrophin gene expression. Here we describe a novel artificial transcription factor, named “UtroUp”, engineered to further improve the DNA-binding specificity. UtroUp has been designed to recognise an extended DNA target sequence on both the human and mouse utrophin gene promoters. The UtroUp DNA-binding domain contains six zinc finger motifs in tandem, which is able to recognise an 18-base-pair DNA target sequence that statistically is present only once in the human genome. To achieve a higher transcriptional activation, we coupled the UtroUp DNA-binding domain with the innovative transcriptional activation domain, which was derived from the multivalent adaptor protein Che-1/AATF. We show that the artificial transcription factor UtroUp, due to its six zinc finger tandem motif, possesses a low dissociation constant that is consistent with a strong affinity/specificity toward its DNA-binding site. When expressed in mammalian cell lines, UtroUp promotes utrophin transcription and efficiently accesses active chromatin promoting accumulation of the acetylated form of histone H3 in the utrophin promoter locus.

**Conclusions:**

This novel artificial molecule may represent an improved platform for the development of future applications in DMD treatment.

## Background

Duchenne Muscular Dystrophy (DMD) is the most common X-linked degenerative muscle disease. The diagnostic marker for DMD is the absence of the cytoskeletal protein dystrophin, which plays a major structural role in muscle by providing stability to the sarcolemma during muscle contractions [1]. DMD still lacks an effective cure; although different therapeutic strategies for DMD are currently being explored [2-6], a variety of drawbacks has significantly delayed their clinical translation. A promising approach for DMD therapy is based on increasing the levels of utrophin, a cytoskeletal protein that is similar to dystrophin and is able to compensate for its absence. Dystrophin and utrophin display a high degree of homology, and both bind members of the dystrophin-associated protein complex (DAPC) [7]. In adult muscle, utrophin is localised preferentially at the neuromuscular junction (NMJ) and myotendinous junctions, while dystrophin is localised along the entire length of the sarcolemma [8]. However, utrophin is also found along the sarcolemma in developing muscle, in regenerating muscle after injury and in mdx (dystrophin-deficient) skeletal muscle [9]. In DMD patients, utrophin is often upregulated, but this upregulation is not sufficient to prevent the progression of muscular dystrophy. Although the adenoviral delivery of utrophin in the mouse model of DMD (*mdx*) and in the dystrophin-deficient dog ameliorates the pathology, the huge size of the utrophin gene is a critical disadvantage [10]. Therefore, studies developing natural or synthetic small molecules that upregulate utrophin could accelerate the clinical translation process [11-17]. To obtain upregulation of utrophin, we have engineered artificial zinc finger-based transcription factors that are capable of binding and activating transcription from promoter “A” of both the human and mouse utrophin genes [18-23]. Zinc finger domains have been shown to be optimal building blocks for generating artificial transcription factors due to their versatility and modularity [24,25]. In particular, a “recognition code” that relates the amino acids of a single zinc finger to its associated DNA target has been utilised as a guide for the DNA binding design [24-30]. Changes in the key amino acid positions (−1, +3 and +6) of the zinc finger alpha-helix alter the DNA-binding specificity of a zinc finger and enable it to bind the programmed DNA-binding site [24]. We generated transgenic mice that specifically over-express an artificial three zinc finger protein in the muscle, named “Jazz”, which is able to specifically upregulate the utrophin gene [20]. Crossing the Jazz transgenic mice with the mouse Duchenne muscular dystrophy mouse model “mdx” results in a strong amelioration of the dystrophic phenotype [22,23]. In a continued attempt to improve the artificial transcription factor’s DNA-binding affinity/specificity, we engineered “UtroUp” that recognises a longer DNA target sequence than its prototype gene Jazz. UtroUp has been designed to target the eighteen-base-pair DNA sequence present in both human and mouse utrophin gene promoters “A”. This target sequence is unique and conserved in both genomes. Here, we show that the artificial transcription factor UtroUp, due to its six zinc finger tandem motif, and its low dissociation constant (Kd) possesses a strong affinity/specificity toward its DNA-binding site. UtroUp, coupled with its activation domain that was derived from the adaptor protein Che-1/AATF [21], efficiently accesses the active chromatin in the utrophin promoter locus and strongly activates utrophin transcription. This novel artificial molecule represents an improved platform for the development of future applications in the DMD gene therapy field.

## Methods

### Constructs

The synthesis of the UtroUp gene was performed by GenScript (New Jersey, USA). The company provided us with the gene cloned into the pUC57 vector at the EcoRI and XhoI restriction sites. We sub-cloned the synthetic gene into pGEX-4T1 bacterial expression vector (GST-UtroUp) and into the pRK5/myc mammalian expression vector, under the control of cytomegalovirus regulatory regions, in fusion with the CJ7 trans-activation domain (CJ7-UtroUp) [21]. CJ7-Jazz molecule was cloned in the same pRK5/myc mammalian expression vector. Note that here, we simply referred to CJ7 as the activation domain, which is one hundred amino acids long and strictly derived from the human Che-1/AATF protein, and not as the entire fusion protein that was described in our previous publication [21].

### Expression of the bacterial recombinant fusion proteins

The recombinant GST-UtroUp protein and the recombinant GST-Jazz protein were expressed in BL21(DE3) host bacteria by an IPTG induction and were purified using glutathione Sepharose 4B beads as previously described [18]. The GST fusion proteins were separated by SDS-PAGE electrophoresis, visualized by Coomassie blue staining and quantified.

### Electrophoretic mobility shift assay (EMSA) and dissociation constant

The EMSA were performed as previously described [18]. The oligonucleotide probes containing one copy of the WT UtroUp DNA target (underlined) were the following: forward primer 5^′^-A TTA AGC CGG GCT GCT GCG GGC TGG GAG TAT GAT CC-3^′^ and reverse primer 5^′^-GG ATC ATA CTC CCA GCC CGC AGC AGC CCG GCT TAA T-3^′^. The mutagenized oligonucleotide probes were: “Mutated” UtroUp forward primer 5^′^-A TTA AGC CGG GCT GCT taa taa TGG GAG TAT GAT CC-3^′^ and reverse primer 5^′^-GG ATC ATA CTC CCA tta tta AGC AGC CCG GCT TAA T-3^′^; “Scrambled” UtroUp forward primer 5^′^-A TTA AGC CGG ACA ACC ATC GAT GTC CGT TAT GAT CC-3^′^ and reverse primer 5^′^-GG ATC ATA ACG GAC ATC GAT GGT TGT CCG GCT TAA T-3^′^. The oligos used in the EMSA were labelled by a T4 polynucleotide kinase. The binding buffer that was used contained 20 mM Hepes pH 7.5, 0.5 mM DTT, 100 mM NaCl, 50 μM ZnCl_2_, 50 μg/ml BSA, 100 ng of poly (dI-dC), 0.05% NP40 and 5% glycerol. Percentage of both inputs: WT and the “6 base pair” mutated DNA target probes shifted at each protein concentration were quantified using ImageJ analysis software and plotted in a graph. To measure and compare the apparent dissociation constant (Kd) of the GST-UtroUp and GST-Jazz complexed with their DNA-target, we performed a series of EMSA with increasing amount of GST-UtroUp (1, 2, 3, 4, 8, 12, 30, 60 nM and 90 nM) or increasing amount of GST-Jazz (5, 8, 16, 32, 64 and 96 nM) incubated with a constant amount of labelled UtroUp-Jazz DNA target, following the procedure previously described [18]. Briefly, the radioactive signals were visualised by autoradiography and quantified by the ImageQuant software (Molecular Dynamics, Sunnyvale, CA) and the data were analysed with the KaleidaGraph programme (Abelbeck Software, Reading, PA). The Kd values were determined as an average of data as mean ± S.D. of five separate experiments.

### Cell lines, transient transfections and reporter gene assay

The human HeLa cell line was grown in Dulbecco’s modified Eagle’s medium (DMEM) (Gibco Corporation, Grand Island, NY, USA) supplemented with 10% foetal calf serum. Transient transfection experiments in the HeLa cell line were carried out using Lipofectin and PLUS reagents (Life Technologies Corporation, Carlsbad, Ca.) according to the manufacturer’s instructions. Cell extracts were prepared and assayed for luciferase (LUC) activity according to the manufacturer’s instructions (Promega, Madison, WI, USA) using a Berthold LB9506 luminometer. The total protein in the extracts was quantified using a Bradford assay, and the LUC activity from equal amounts of protein was determined and normalised for beta-galactosidase activity.

### Chromatin immunoprecipitation assay (ChIP)

Chromatin Immunoprecipitation was performed using a ChIP assay kit according to the manufacturer’s instructions (Upstate Biotechnology, Charlottesville, VA, USA). Approximately 8 million cells were cross-linked with 1% formaldehyde for 10 min at 37°C and then lysed. The cell lysate was sonicated on ice, which resulted in DNA fragments of approximately 500 bp in length. Equal amounts of chromatin from each sample were immunoprecipitated over night with either an anti-acetylated-Histone H3 rabbit polyclonal antibody (Upstate Biotechnology) or anti-myc 9E10 monoclonal antibody. DNA representing 0.005% of the sonicated chromatin solution (input) and 10% of the immunoprecipitated sonicated chromatin solution were amplified using the following primers: human utrophin promoter region primers forward 5^′^-CGGCACGCACGGTTCACTCTGGAGCGC-3^′^ and reverse 5^′^-CAGCAACTTTGTTCCGGAAGATCAGCC-3^′^; human thymidine kinase promoter region–specific primers forward 5^′^-GCCCCTTTAAACTTGGTGGGCGG-3^′^ and reverse 5^′^-TTGCGCCTCCGGGAAGTTCACG-3^′^; human dystrophin promoter region–specific primers forward 5^′^-GTGTTTTAAGAATTGGCACCAG-3^′^ and reverse 5^′^-AGTCTGAATAAGAGAAGCAGCA -3^′^; human chromosome 16 region–specific primers forward 5^′^-AGGACCACTCGCTGGGTAAGCA-3^′^ and reverse 5^′^-CGCGGAGGGTGACATGGGGT-3^′^; human chromosome 17 region–specific primers forward 5^′^-ACCTGTGTGTGGGTGGTGAGA-3^′^ and reverse 5^′^-CTGTAGGGCCCCAGGCACCAT-3^′^. The DNA sequence of the potential off-target site present in chromosome 16 is: 5^′^-GCTGCTGgGGGCTGGGgc-3^′^, with 15/18 matches with UtroUp target sequence. The DNA sequence of the potential off-target present in chromosome 17 is: 5^′^-GCTGCTGCGGGCTGGGga-3^′^, with 16/18 matches with UtroUp Target sequence. The PCR conditions were the following: 30 cycles at 95°C for 45 s, 62°C for 30 s, 72°C for 30 s and a final extension at 72°C for 5 min. Quantification of each chromatin immunoprecipitation (ChIP) experiment was performed using ImageJ software and plotted in a graph.

### RNA extraction, reverse transcriptase reaction and real-time PCR

Total RNA was extracted from HeLa cells expressing either CJ7-UtroUp or the negative control pRK5 using the TRIzol reagent according to the manufacturer’s instructions (Life Technologies). Two μg of RNA was reverse transcribed using oligo (dT)_12–18_ primers and Superscript II (Life Technologies) in a final volume of 20 μl at 42°C for 50 min. A real-time PCR assay was performed in a 96-well format using the ABI Prism 7000 Sequence Detection System (Applied Biosystems, Foster City, CA). Primers and probes for utrophin (target gene) and for GAPDH (housekeeping gene) were purchased as TaqMan Gene Expression Assays (Applied Biosystems). PCR mixtures containing the cDNA template, the TaqMan Universal PCR master mix (Applied Biosystems) and the primers/probes in a final volume of 25 μl were analysed in triplicate using the following conditions: an incubation at 50°C for 2 min, denaturing at 95°C for 10 min and then 40 cycles of the amplification step at 95°C for 15 s and 60°C for 1 min. For each gene amplification, a standard curve was generated using serial dilutions (200, 40, 8, 1.6 and 0.32 ng) of cDNA from the HeLa cells (negative control). The results were analysed using the Applied Biosystems analysis software. The data are expressed as the ratio between utrophin and GAPDH mRNA expression.

### Western blot

Proteins were extracted from HeLa cells using the following lysis buffer (50 mM Tris pH 7.4, 50 mM EDTA, 250 mM NaCl, 50 mM NaF, 0.1% Triton X-100, 0.1 mM NaVO_4_ and 0.5% glycerol) containing protease inhibitors. Equal amounts of total protein (30–40 μg) were separated by SDS-PAGE electrophoresis, transferred to membranes and immunoblotted with the following antibodies: mouse monoclonal anti-myc 9E10, mouse monoclonal anti-alpha tubulin (Sigma Corporation, St. Louis, Missouri, USA), mouse monoclonal anti-utrophin (NOVOCASTRA, Newcastle, UK), rabbit polyclonal anti-laminin (Sigma Corporation, St. Louis, Missouri, USA), mouse monoclonal anti-beta-Actin (Sigma Corporation, St. Louis, Missouri, USA), rabbit polyclonal anti-Sp1 (Santa Cruz, Santa Cruz, CA). Densitometric analysis of utrophin protein level was performed using ImageJ analysis software.

## Results and discussion

**Figure 1:**
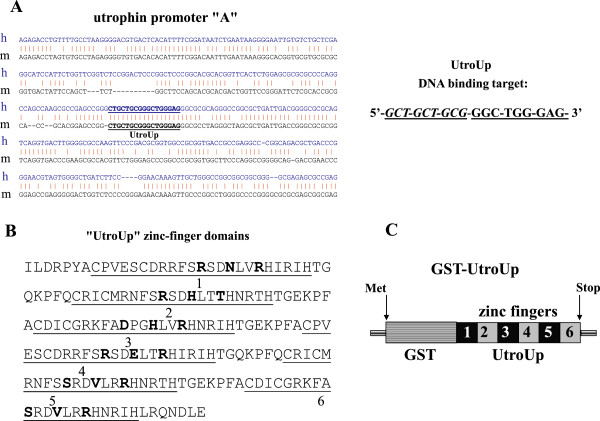
**A graphical representation of the human and mouse utrophin promoters and the amino acid sequences of the six zinc finger UtroUp peptide (GenBank JQ073900). A:** On the left is shown an alignment of DNA sequences of the human and mouse utrophin promoter “A”. The UtroUp DNA target sequence, which is 18 base pairs long, is underlined. The DNA target sequence is 100% conserved between humans and mice. On the right is shown the 18-base-pair UtroUp DNA target sequence. The portion of the Jazz DNA target sequence is indicated in italics. **B:** The amino acid sequence of the synthetic six zinc finger DNA-binding domain of the UtroUp peptide. Each zinc finger domain in the tandem array is underlined and numbered. The amino acid residues in the α-helix (−1, +3 and +6), which are key for the DNA-binding specificity, are indicated in bold. The zinc finger domains 4, 5 and 6 are inherited by the Jazz peptide. **C:** A schematic representation of the bacterially produced UtroUp peptide fused to glutathione S-transferase (GST).

One central point in the designing of synthetic zinc finger peptides is to increase their specificity toward the desired DNA target [25,31]. The simplest approach for success is to increase the length of the target sequence to decrease its frequency in the genome. By assuming a random base distribution, an 18-base-pair DNA target sequence is statistically predicted to be unique in the haploid human genome. We chose the following DNA target sequence: 5^′^- GCT GCT GCG GGC TGG GAG- 3^′^, that is present only once in both human and mouse genomes, in the utrophin gene promoter “A” (Figure 1A). In order to target this 18-base-pair DNA sequence we designed and constructed a novel artificial gene named “UtroUp” containing six zinc finger domains in a tandem array. To be able to test the therapeutic potential of our artificial transcription factor in a dystrophic mouse model (mdx), we chose a DNA target sequence in a region that was completely conserved in both the human and the mouse utrophin promoter “A” (Figure 1A). This region is located near several natural transcription factor DNA-binding sites in a chromatin-accessible region [18]. UtroUp, with its six zinc finger domains, is an extended version of the previously engineered three zinc finger transcription factor Jazz [18,20-22]. At its 5^′^ end, the UtroUp DNA target sequence comprises 9 base pairs of the DNA target sequence of Jazz, as indicated in italics in Figure 1A (right side). Figure 1B shows the amino acid sequence of the UtroUp peptide. The first three UtroUp zinc fingers have been newly designed, engineered and selected, while the last three are coincident with the previously engineered Jazz zinc finger domains. The amino acids responsible for the specific nucleotide contact in each zinc finger (alpha helix, positions −1, +3 and +6) have been chosen on the basis of the available “recognition code” that combines the zinc finger primary structure and potential DNA-binding sites [18,24,28-30]. We have generated UtroUp by coupling the available recognition code with DNA binding performance selection evaluated by EMSA [18]. It is important to note that the zinc finger peptide binds the DNA target sequence in an anti-parallel manner. To test the UtroUp DNA-binding ability, we produced and purified UtroUp, which was fused to a glutathione S-transferase (GST) domain (GST-UtroUp), in bacteria (Figure 1C). The affinity/specificity of GST-UtroUp toward its putative DNA target sequence was evaluated by electrophoretic mobility shift assays (EMSA).

**Figure 2:**
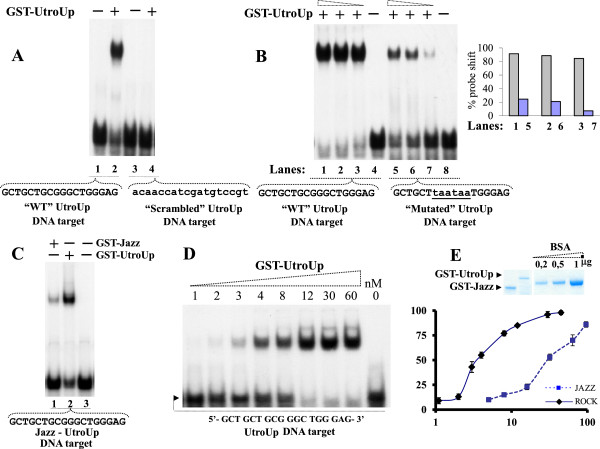
**The UtroUp DNA-binding affinity/specificity analysed by EMSA. A:** An equal amount of the GST-UtroUp protein (14 nM) was incubated with an equal amount of either the labelled wild-type DNA target (lanes 1–2) or with the labelled scrambled DNA target (lanes 3–4). **B:** (Left) An increasing amount of the GST-UtroUp protein (20, 40, 80 nM) was incubated with an equal amount of either the labelled wild-type DNA target (lanes 1–4) or with the labelled mutant DNA target (lanes 5–8). The two mutated triplets in the DNA target are indicated by lowercase characters. (Right) Histogram shows a percentage of probes (WT and Mutated) shifted with increasing amounts of GST-UtroUp protein. **C:** An equal amount (12 nM), of the GST-UtroUp protein (lane 2) and GST-Jazz protein (lane 1) were incubated with an equal amount of labelled UtroUp-Jazz DNA target. **D:** An EMSA prototype performed for the determination of the dissociation constant (Kd). An increasing amount of GST-UtroUp protein (1, 2, 3, 4, 8, 12, 30, and 60 nM) was complexed with the labelled UtroUp DNA target. **E:** (Top) Quality control by Coomassie blue staining of purified/eluted GST-UtroUp and GST-Jazz fusion proteins compared with purified commercial bovine serum albumin. (Bottom) The Kd values of the GST-UtroUp/DNA-target and GST-Jazz/DNA complexes correspond approximately to 3,5 nM and 32 nM respectively. The protein concentration is expressed in nM.

**Figure 3:**
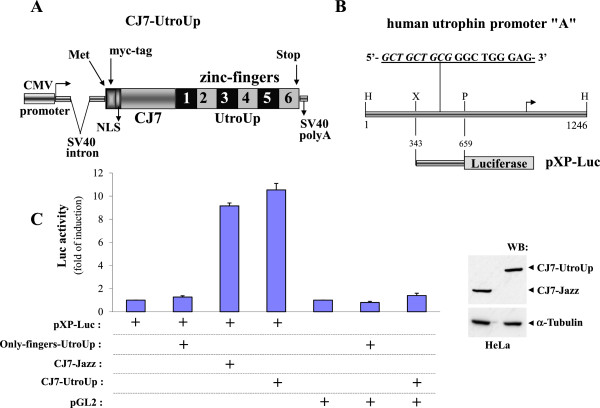
**The trans-activation of the human utrophin promoter “A”. A:** Schematic representation of the CJ7-UtroUp chimera in the mammalian expression vector. **B:** A graphical representation of the human utrophin promoter “A”. The pXP-Luc construct consists of a minimal region of the human utrophin promoter “A” containing the 18-base-pair UtroUp target DNA-binding sequence (indicated in italicised and underlined characters) that was cloned upstream of the reporter gene (Luciferase). **C:** (Left) A histogram shows the fold of induction of the pXP-Luc utrophin promoter “A” construct or the control reporter pGL2-Promoter (Promega) that was obtained upon co-transfection with the finger region-only UtroUp, or the CJ7-UtroUp or the CJ7-Jazz in HeLa cells. The data are presented as the means ± S.D. of three independent experiments that were performed in triplicate. (Rigth) Western blot analysis cell lysates from HeLa cells transiently cotransfected with pXP-Luc construct and the indicated CJ7-UtroUp or CJ7-Jazz expression vectors. The membrane was incubated with the anti-myc tag antibody and the anti-alpha tubulin antibody.

As shown in Figure 2A a marked shift is observed when GST-UtroUp is incubated with the wild type (WT) oligonucleotide probe containing UtroUp DNA-binding site (lane 2), while no shift is revealed in presence of the “Scrambled” oligonucleotide probe (lane 4). Then, to test the overall binding activity of UtroUp, we concentrated on the DNA binding contribution of the two crucial central zinc fingers 3 and 4. We compared, in an EMSA assay, increasing amount of GST-UtroUp with either WT oligonucleotide target or centrally mutated oligonucleotide target. As shown in Figure 2B, a strong saturated shift signal is observed even at the lowest concentration of GST-UtroUp protein in the presence of the wild type oligonucleotide target (lanes 1–3), while the GST-UtroUp shift appears dramatically compromised in the presence of six base pairs mutated oligonucleotide DNA target (lanes 5–7) (see quantification analysis, Figure 2, panel B right). Importantly, comparing GST-UtroUp and GST-Jazz performance in the same EMSA, the DNA target shift in presence of GST-UtroUp is clearly stronger than the shift obtained with GST-Jazz (Figure 2C). Then, to measure the affinity of the GST-UtroUp toward its DNA target sequence, the dissociation constant (Kd) was determined by EMSA performed using increasing amounts of GST-UtroUp protein combined with a constant amount of labelled UtroUp DNA oligonucleotide target (Figure 2D). In our experimental conditions, we obtained for UtroUp a Kd of approximately 3,5 nM (Figure 2E). The UtroUp Kd value is lower than the Kd values measured for our artificial transcription factors that contain three or four zinc finger domains [18,19], indicating a tighter binding of UtroUp to the DNA target sequence. In particular, UtroUp Kd value is about ten-fold lower than the Kd value of its progenitor Jazz (32nM), as shown in the EMSA derived graph in Figure 2E. Based on the encouraging UtroUp Kd data, we characterised the biological activity of UtroUp in the human HeLa cell line, as both a simple six zinc finger peptide (UtroUp) and as a fusion with the strong “CJ7” activation domain, which resulted in the CJ7-UtroUp molecule (Figure 3A) [21]. The CJ7 transcriptional activation domain was derived from the regulatory multivalent adaptor Che-1/AATF [32-34]. The CJ7 domain is 100 amino acids in size, and it is a stronger activator than the canonical herpes simplex virus-derived Vp16 activation domain [21]. We previously showed that the CJ7 domain in fusion with the synthetic three zinc finger Jazz protein efficiently promotes transcription and the accumulation of the acetylated form of histone H3 on the genomic utrophin promoter locus [21]. To evaluate the relative transcriptional activity of the CJ7-UtroUp and the CJ7-Jazz artificial transcription factors [21], we separately transfected, into HeLa cells, the constructs expressing the two molecules and the construct expressing only the UtroUp zinc finger DNA binding domain (as a control) together with the luciferase reporter construct pXP-Luc [35], which contained the utrophin promoter “A” region including the 18-base-pair UtroUp target sequence (Figure 3B).

**Figure 4:**
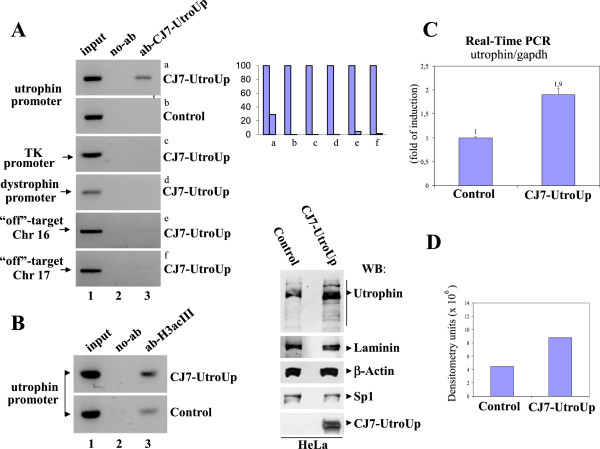
**The binding and upregulation of the endogenous utrophin gene. A:** (Left) The ChIP assay was performed in transfected HeLa cells (with either an empty control vector or with the CJ7-UtroUp expression vector) using myc-tag monoclonal antibody/protein G-agarose beads. The immunoprecipitates from each sample were analysed by PCR performed using primers specific for the human utrophin promoter “A”. PCRs of the promoters of the unrelated thymidine kinase and dystrophin genes and PCRs of specific regions on the chromosomes 16 and 17 were included as controls. A sample representing the linear amplification of the total input chromatin (input) was included (lane 1). (Right) Histogram shows quantification of ChIP-PCR bands presented as fraction of the input. **B:** ChIP was performed in transfected HeLa cells (with either the CJ7-UtroUp expression vector or with an empty control vector) using anti-acetylated histone H3 rabbit polyclonal antibody/protein G-agarose beads. Immunoprecipitates from each sample were analyzed by PCR performed using primers specific for human utrophin promoter “A”. **C:** The analysis by real-time PCR of the utrophin gene mRNA expression in HeLa cells transfected with the CJ7-UtroUp or control vectors. The ratio between the utrophin and GAPDH gene expression is shown as means ± S.D. from three independent experiments that were performed in triplicate. **D:** (Left) Western blot analysis of total protein lysates from HeLa cells transfected with either empty vector (Control) or with the CJ7-UtroUp expression vector. The membrane was incubated with the indicated antibodies. (Right) Histogram shows utrophin expression evaluated by densitometric analysis.

As shown in the histogram in Figure 3C, the analysis of the luciferase activity revealed a ten to eleven fold induction of the pXP-Luc construct in the presence of CJ7-UtroUp. This strong activation appears to be highly specific, and it is even stronger than the activation obtained using the prototype CJ7-Jazz [21], taking into account a comparable expression of CJ7-UtroUp and CJ7-Jazz proteins, as shown in the western blot (Figure 3C). To verify whether the six zinc finger protein CJ7-UtroUp accesses the utrophin promoter locus in the chromatin infrastructure, we performed chromatin immunoprecipitation experiments (ChIPs). As shown in Figure 4A, ChIP experiments demonstrated that CJ7-UtroUp accesses the genomic utrophin promoter A locus in an efficient way. To verify CJ7-UtroUp specificity toward the genomic utrophin promoter locus, we tested in the same ChIP experiments unrelated gene-promoters and potential UtroUp genomic “off-target” sites. A BLAST search indicated that the 18 base pairs UtroUp target sequence is present only once in both human and mouse genomes. As expected, the BLAST search pointed out several potential chromosomal off-target sites containing a number of nucleotide mismatches within the 18 base pairs UtroUp target sequence. In particular, we tested two potential off-target sites present in loci on human chromosome 16 and chromosome 17, carrying 15 base pair matches out of 18 and 16 base pair matches out of 18 respectively (see material and method section). A quantification analysis of all ChIP/PCR bands is reported in Figure 4A (right side). All these ChIP data taken together indicate a high specificity of CJ7-UtroUp protein toward its DNA target in the utrophin promoter A. Moreover as shown in Figure 4B a ChIP assay demonstrated that CJ7-UtroUp is able to promote accumulation of the acetylated form of histone H3. Hyperacetylation correlates with transcriptional activation and indicates that histone-modifying activities [36,37] could contribute to the transcriptional control induced by CJ7-UtroUp. Next, we determined the level of endogenous utrophin upregulation that was induced by the expression of CJ7-UtroUp. To this end, we performed a quantitative analysis of the utrophin mRNA levels using real-time PCR. As shown in Figure 4C, we observed an approximate 2.0-fold increase in utrophin transcription level in the presence of CJ7-UtroUp compared to the control sample. Additionally, to determine whether the changes in the utrophin mRNA expression levels, which were induced by the presence of CJ7-UtroUp, were consistent with changes in the utrophin protein level, we performed western blot analysis using an anti-utrophin antibody. As shown in Figure 4D, we observed a perfect correlation between the upregulation of the utrophin mRNA and the utrophin protein. In addition, CJ7-UtroUp appears to mimic the natural transcriptional regulatory mechanisms, which give rise to an increase in all of the different utrophin isoforms, as shown by the utrophin protein pattern in western blot.

The synthetic transcription factor Jazz [18], with its three zinc finger domains, binds a 9-base-pair DNA sequence. Bagly [19], the first Jazz variation, has four zinc finger domains and binds a 12-base-pair DNA sequence. As described here, UtroUp, with its six zinc finger domains, binds an 18-base-pair DNA sequence. Notably, a 9-base-pair DNA sequence is statistically present in the human genome (≅ 3.5 × 10^9^ bp) approximately 1.3 × 10^4^ times, a 12-base-pair sequence is present 210 times and an 18-base-pair sequence is present only once [31]. Moreover, when fused with our strong activation domain CJ7, UtroUp specifically and efficiently upregulates the utrophin gene expression.

## Conclusions

In an attempt to improve the efficacy of our artificial transcription factors, engineered to reprogram the expression of the utrophin gene, we focused on tuning both DNA-binding affinity/specificity and transcriptional performances. Our novel artificial transcription factor, CJ7-UtroUp, represents a good compromise between small size, high DNA target affinity/specificity and efficient transcriptional activation. The small size makes UtroUp particular suitable for viral vector delivery and the possibility of targeting a unique site in the genome extremely reduces off-target effects on global transcription. UtroUp and its upcoming variations may represent an important platform for the development of future applications in the DMD gene therapy field. Moreover, human myogenic cell lines and mouse models expressing UtroUp can offer an unique model system for screening novel compounds that are able to upregulate the utrophin gene for DMD treatment.

Importantly, our results contribute to demonstrate that artificial zinc finger transcription factors may be a class of therapeutic reagents for treatment of crucial inherited diseases [38].

## Abbreviations

DMD: Duchenne Muscular Dystrophy; DAPC: Dystrophin-associated protein complex; NMJ: Neuromuscular junction; GST: Glutathione S-transferase; EMSA: Electrophoretic mobility shift assays; Kd: Dissociation constant; ChIP: Chromatin immunoprecipitation; BLAST: Basic Local Alignment Search Tool.

## Competing interests

The authors declare that they have no competing interests.

## Authors’ contributions

NC and CP conceived and designed the experiments. AO, CiP, NC, CP, GS, LM and EM performed the experiments. AO, CiP, NC and CP analyzed the data. NC and CP wrote the manuscript. All the authors read and approved the final manuscript.

## References

[B1] McNally EM Pytel P Muscle Disease: The Muscular Dystrophies Annu Rev Pathol Mech Dis 2007 2 87 109 10.1146/annurev.pathol.2.010506.091936 18039094

[B2] Fairclough RJ Bareja A Davies KE Progress in therapy for Duchenne muscular dystrophy Exp Physiol 2011 96 11 1101 1113 10.1113/expphysiol.2010.053025 21804140

[B3] Pichavant C Aartsma-Rus A Clemens PR Davies KE Dickson G Current status of pharmaceutical and genetic therapeutic approaches to treat DMD Mol Ther 2011 19 5 830 840 10.1038/mt.2011.59 21468001 PMC3098643

[B4] Goyenvalle A Seto JT Davies KE Chamberlain J Therapeutic approaches to muscular dystrophy Hum Mol Genet 2011 20 R1 R69 78 10.1093/hmg/ddr105 21436158 PMC3095062

[B5] Meng J Muntoni F Morgan JE Stem cells to treat muscular dystrophies - where are we? Neuromuscul Disord 2011 21 1 4 12 10.1016/j.nmd.2010.10.004 21055938

[B6] Cossu G Sampaolesi M New therapies for Duchenne muscular dystrophy: challenges, prospects and clinical trials Trends Mol Med 2007 13 12 520 526 10.1016/j.molmed.2007.10.003 17983835

[B7] Blake DJ Weir A Newey SE Davies KE Function and genetics of dystrophin and dystrophin-related proteins in muscle Physiol Rev 2002 82 291 329 11917091 10.1152/physrev.00028.2001

[B8] Ohlendieck K Ervasti JM Matsumura K Kahl SD Leveille CJ Dystrophin-related protein is localized to neuromuscular junctions of adult skeletal muscle Neuron 1991 7 499 508 10.1016/0896-6273(91)90301-F 1654951

[B9] Gramolino AO Karpati G Jasmin BJ Discordant expression of utrophin and its transcript in human and mouse skeletal muscles J Neuropathol Exp Neurol 1999 58 235 244 10.1097/00005072-199903000-00003 10197815

[B10] Nowak KJ Davies KE Duchenne muscular dystrophy and dystrophin: pathogenesis and opportunities for treatment EMBO Rep 2004 5 9 872 876 10.1038/sj.embor.7400221 15470384 PMC1299132

[B11] Khurana T Davies KE Pharmacological strategies for muscular dystrophy Nat Rev Drug Discov 2003 2 379 390 10.1038/nrd1085 12750741

[B12] Miura P Jasmin BJ Utrophin upregulation for treating Duchenne or Becker muscular dystrophy: how close are we? Trends Mol Med 2006 3 122 129 16443393 10.1016/j.molmed.2006.01.002

[B13] Stocksley MA Chakkalakal JV Bradford A Miura P De Repentigny Y A 1.3 kb promoter fragment confers spatial and temporal expression of utrophin A mRNA in mouse skeletal muscle fibers Neuromuscul Disord 2005 15 6 437 449 10.1016/j.nmd.2005.03.008 15907291

[B14] Basu U Gyrd-Hansen M Baby SM Lozynska O Krag TO Heregulin-induced epigenetic regulation of the utrophin-A promoter FEBS Lett 2007 4;581 22 4153 4158 10.1016/j.febslet.2007.07.021 PMC2699486 17692845

[B15] Perkins KJ Basu U Budak MT Ketterer C Baby SM Ets-2 repressor factor silences extrasynaptic utrophin by N-box mediated repression in skeletal muscle Mol Biol Cell 2007 18 8 2864 2872 10.1091/mbc.E06-12-1069 17507653 PMC1949368

[B16] Tinsley JM Fairclough RJ Storer R Wilkes FJ Potter AC Daily treatment with SMTC1100, a novel small molecule utrophin upregulator, dramatically reduces the dystrophic symptoms in the mdx mouse PLoS One 2011 6 5 e19189 10.1371/journal.pone.0019189 21573153 PMC3089598

[B17] Amenta AR Yilmaz A Bogdanovich S McKechnie BA Abedi M Biglycan recruits utrophin to the sarcolemma and counters dystrophic pathology in mdx mice Proc Natl Acad Sci U S A 2011 108 2 762 767 10.1073/pnas.1013067108 21187385 PMC3021068

[B18] Corbi N Libri V Fanciulli M Tinsley JM Davies KE Passananti C The artificial zinc finger coding gene ‘Jazz’ binds the utrophin promoter and activates transcription Gene Ther 2000 7 1076 1083 10.1038/sj.gt.3301204 10871758

[B19] Onori A Desantis A Buontempo S Di Certo MG Fanciulli M The artificial 4-zinc-finger Bagly binds human utrophin promoter A at the endogenous chromosomal site and activates transcription Biochem Cell Biol 2007 85 3 358 365 10.1139/O07-015 17612630

[B20] Mattei E Corbi N Di Certo MG Strimpakos G Severini C Utrophin up-regulation by an artificial transcription factor in transgenic mice PLoS One 2007 22; 2 1 e774 10.1371/journal.pone.0000774 PMC1942121 17712422

[B21] Desantis A Onori A Di Certo MG Mattei E Fanciulli M Novel activation domain derived from Che-1 cofactor coupled with the artificial protein Jazz drives utrophin upregulation Neuromuscul Disord 2009 2 158 162 19162479 10.1016/j.nmd.2008.11.005

[B22] Di Certo MG Corbi N Strimpakos G Onori A Luvisetto S The artificial gene Jazz, a transcriptional regulator of utrophin, corrects the dystrophic pathology in mdx mice Hum Mol Genet 2010 19 5 752 760 10.1093/hmg/ddp539 19965907

[B23] Passananti C Corbi N Onori A Di Certo MG Mattei E Transgenic mice expressing an artificial zinc finger regulator targeting an endogenous gene Methods Mol Biol 2010 649 183 206 10.1007/978-1-60761-753-2_11 20680835

[B24] Corbi N Libri V Onori A Passananti C Synthetic zinc finger peptides: old and novel applications Biochem Cell Biol 2004 82 428 36 10.1139/o04-047 15284895

[B25] Klug A The discovery of zinc fingers and their development for practical applications in gene regulation and genome manipulation Q Rev Biophys 2010 43 1 1 21 10.1017/S0033583510000089 20478078

[B26] ChooY K A: Physical basis of a protein-DNA recognition code Curr Opin Struct Biol 1997 7 1 117 125 10.1016/S0959-440X(97)80015-2 9032060

[B27] Pabo CO Peisach E Grant RA Design and selection of novel Cys2His2 zinc finger proteins Annu Rev Biochem 2001 70 313 340 10.1146/annurev.biochem.70.1.313 11395410

[B28] Segal DJ Barbas CF 3rd Custom DNA-binding proteins come of age: polydactyl zinc-finger proteins Curr Opin Biotechnol 2001 12 6 632 637 10.1016/S0958-1669(01)00272-5 11849947

[B29] Klug A The discovery of zinc fingers and their applications in gene regulation and genome manipulation Annu Rev Biochem 2010 79 213 31 10.1146/annurev-biochem-010909-095056 20192761

[B30] Bhakta MS Segal DJ The generation of zinc finger proteins by modular assembly Methods Mol Biol 2010 649 3 30 10.1007/978-1-60761-753-2_1 20680825 PMC4323424

[B31] Sera T Zinc-finger-based artificial transcription factors and their applications Adv Drug Deliv Rev 2009 61 7–8 513 526 19394375 10.1016/j.addr.2009.03.012

[B32] Fanciulli M Bruno T Di Padova M De Angelis R Iezzi S Iacobini C Identification of a novel partner of RNA polymerase II subunit 11, Che-1, which interacts with and affects the growth suppression function of Rb FASEB J 2000 14 7 904 912 10783144 10.1096/fasebj.14.7.904

[B33] Passananti C Floridi A Fanciulli M Che-1/AATF, a multivalent adaptor connecting transcriptional regulation, checkpoint control, and apoptosis Biochem Cell Biol 2007 85 4 477 83 10.1139/O07-062 17713582

[B34] Passananti C Fanciulli M The anti-apoptotic factor Che-1/AATF links transcriptional regulation, cell cycle control, and DNA damage response Cell Div 2007 16 2 21 17634135 10.1186/1747-1028-2-21 PMC1948887

[B35] Dennis CL Tinsley JM Deconinck AE Davies KE Molecular and functional analysis of the utrophin promoter Nucleic Acids Res 1996 24 1646 1652 10.1093/nar/24.9.1646 8649981 PMC145847

[B36] Verschure PJ Visser AE Rots MG Step out of the groove: epigenetic gene control systems and engineered transcription factors Adv Genet 2006 56 163 204 16735158 10.1016/S0065-2660(06)56005-5

[B37] Beltran AS Blancafort P Remodeling genomes with artificial transcription factors (ATFs) Methods Mol Biol 2010 649 163 182 10.1007/978-1-60761-753-2_10 20680834

[B38] Costa FC Fedosyuk H Neades R de Los Rios JB Peterson KR Barbas CF 3rd Induction of Fetal Hemoglobin In Vivo Mediated by a Synthetic γ-Globin Zinc Finger Activator Anemia 2012 2012 507894 10.1155/2012/507894 22778925 PMC3384929

